# General and Local Treatment of Inflammation

**Published:** 1868-01

**Authors:** J. F. Miner


BUFFALO

### Medical and Surgical Journal.

VOL. VII. JANUARY, 1868. No. 6.

### Original Communications.

ART. I.—*General and Local Treatment of Inflammation.* By J. F. Miner, M. D.

Only the most general rules and principles can be with any propriety proposed in the treatment of a condition which differs so much according to age, cause, location, constitution of the patient, and other influences; yet there are some general considerations which are worthy of attention and may be proposed with advantage. The practice of to-day is nearly as diversified and contradictory as ever, and very little has yet been gained towards uniformity and harmony, though the most wonderful changes and revolutious have taken place, and the most astonishing progress made in discovering the pathological changes and inherent tendencies in that condition of disease which we call inflammation. A very large portion of the time and attention of the general practitioner is expended in the management of affections essentially inflammatory in character, and from the extent and frequency of this pathological condition, if from no other consideration, it may be interesting to consider, what conclusions are best sustained by modern investigation, and what of the formerly received views are shown to be unfounded. This is a subject upon which volumes have been written and volumes more are yet to be written, but condensed is capable of expression in fewer words.

The local deviations from health, which we designate as inflammation, are pretty well understood. The increased flow of blood, distension of vessels and stagnation in the blood current, the effused products of this condition and their effects upon the various organs and tissues according to their functions and structures have been faithfully studied, and but few differences of opinion at present prevail upon these points, while the causes of this action and the best modes of controlling it, are perhaps as undetermined as ever, or at least as great differcnces of opinion now prevail as at any period in the history of medicine. Perhaps it will not be far from the truth to say, that our present knowledge of inflammation does not permit us to know the causes and many of the changes in nutrition, circulation and other processes essential to, and comprising that condition which constitutes inflammation; that though many plausible theories have been invented, still the exact truth may not yet have been discovered.

These questions are not now to be discussed, but the single and practical one of treatment. What medicines, if any, are known to exert any controlling influence in this common condition of disease, and what other measures, not strictly medicinal, are shown to possess curative or controlling power? A great many remedies have been said possess such properties, and it is an interesting and practical question to inquire, how far such statements are sustained by adequate evidence? The author might undertake the answer to these questions with embarrassment, if the accusation of suffering under "*surgical hallucination*" was expected to greatly influence his readers in their estimate of his knowledge of the value of medicine in the treatment of disease; but as he has exclusively devoted himself for nearly a quarter of a century to the study and practice of medicine, at all times with ample opportunities of observation, it can hardly be supposed that any surgical tastes he may have acquired, could pervert or diminish his apprehension of medical truth. This fact also justifies, in some measure, the expression of personal opinions, since to recite only the views and conclusions of others adds nothing to the amount of positive testimony, and involves the necessity of much writing, to express what had been proposed to be embodied, as far as possible, in few words.

It is to be constantly remembered in the treatment of inflammation that there is a tendency to recovery, which is a part of its natural history, and that in the vast majority of instances this result is attained without assistance from drugs. In inflammation of the lung, the disease passes through its various stages giving out its characteristic products, and terminating in perfect recovery without the administration of medicines considered as curative, indeed without any treatment whatever. The same is observed in inflammation of all other structures, both of internal and external organs. This fact should be so well understood and so constantly acted upon as to require no further repetition, but after all it is liable to be forgotten. This central truth was for a long time almost wholly overlooked, and all inflammatory and febrile diseases subjected to the influence of general blood-letting, which was supposed by many, as capable, if employed early enough and freely enough, of *control* in inflammation.

It is no part of the present purpose to discuss the question of general blood-letting, a subject which, if really commenced, is nearly without end. That inflammation is more often a condition of debility, rather than otherwise, and does not require or bear general blood-letting is the present prevailing opinion, modified by exceptions and subject perhaps to some qualifications.

To come, then, directly to a consideration of the medicines reputed as exercising control over inflammatory diseases, reserving the external remedies, supposed capable of producing this effect for later disposal, mercury must of necessity be expected to stand at the head of the list; unpleasant as it is, to speak of his medicinal majesty, especially if it is desirable to do so in a summary manner.

*Mercury* was for a long time, with wonderful unanimity of opinion, regarded as capable of exercising great influence over inflammations of all varieties, especially of the serous membranes, and in pneumonitis, pleuritis, pericarditis and peritonitis its administration was universal and its necessity undoubted. A change has now taken place, so great, that mercury is no longer administered in these diseases with such view, and its use is restricted to the treatment of syphilitic inflammation affecting the eye, larynx and skin, and to its employment as a cathartic. That mercury has controlling influence over secondary syphilitic disease, is very well established, but it is not a specific for this disease, and is not essential in its management. Syphilitic iritis often appears greatly benefited by this remedy, but many cases are successfully treated without it. The real or apparent efficacy of mercury in syphilitic iritis, contributed considerable to the confidence in it, for inflammations generally, since the diseased condition could be clearly seen through the cornea, and its increase or disappearance easily noted. It was well calculated to mislead, and it is not remarkable that it required time and more careful observation to determine its real value and import. Lymph effused into the iris or upon its surface, often disappears rapidly under the influence of mercury, and the other symptoms of syphilitic disease abate as rapidly, but the same disappearance of this disease in the iris is observed without mercury, and the idea that in all cases of effusion in simple or specific inflammation, mercury is to be administered, is wholly opposed to the better doctrines of present practice. Mercury then, in brief, is not a remedy for inflammation in general, though its virtues in appropriate cases is not to be lost sight of, while it is nearly restricted to specific forms of disease, and to its uses as a cathartic. It is valuable as a local remedy and in some diseases of the skin.

*Antimony* is also a remedy supposed to be of value in inflammation. In bronchitis, laryngitis, and indeed in all inflammations of the mucous membranes, it has been regarded as an important remedy. It diminishes the force of the heart's action, produces nausea and vomiting, probably promotes secretion from the mucous membranes and may have its uses in some very acute forms of inflammation. It is apt to induce irritation of the stomach and bowels, greatly reduces the strength, and in young children should be generally avoided. Its value in cases of inflammation of internal organs is not clearly established; pneumonia, pleurisy and bronchitis. are managed more safely and certainly in almost all instances without the administration of this drug.

*Ipecacuanha* appears valuable in inflammation of mucous membranes, and is not so depressing and objectionable as antimony; it has undoubted influence, and is of value especially in combination with other remedies. *Squill*, *senega*, *blood-root*, *lobelia*, etc., etc., are remedies of the same class; inferior in most respects and require no separate mention.

*Opium* and its principles and compounds comprises the most important known remedy in the treatment of inflammation; in many forms it is directly curative, and in nearly all, paliative in greater or less degree. It is designed to avoid the speculative part of the subject, but in considering the wonderful effects of anodynes over inflammation it cannot be amiss to suggest that this action appears to be through the nervous system, and so strongly shows the power of nervous influence upon inflammation, that there are not wanting careful observers who believe the causes of inflammation to consist wholly or in part in perverted nervous action. However this may be, opium more certainly than any other remedy, if rightly prescribed, exercises not only a curative but a paliative influence; quiets the pain, regulates the circulation, procures sleep, and may be chiefly relied upon in most forms of the disease. Opium is regarded by many as inadmissible in inflammatory diseases of the brain. That there are conditions of disease within the cranium which do not require opium is certain; the coma from pressure of effusion should not be increased or complicated by narcotism; the increased fullness of the vessels, as in apoplexy or preceding the appearance of the disease, may contra indicate opium; but it is wide of the truth to say, that inflammation within the cranium is always or generally increased, or its evil effects augmented by the administration of this drug; on the other hand it is often useful, and its beneficial effects are as manifest and unmistakable as in inflammations elsewhere. Opium, in its various forms, is of more value in the treatment of inflammatory diseases than all other medicinal substances combined, and correct appreciation of its value, and full knowledge of the indications for its employment are essential and absolutely indispensable to the best management of inflammation. In pleurisy, peritonitis, pericarditis and inflammations of serous membranes generally, it may be made to supercede and remove the necessity for depletion, or, is of great value after venesection, should this remedy be thought necessary. In inflammation of mucous surfaces it is also of equal or even greater value, in many is directly curative, in all paliative. It may be misused in pneumonia and in bronchitis, where the accumulation of mucus is to be removed by coughing, but its judicious employment is attended with the greatest advantage. Too great care cannot be observed in its employment with infants and very young children.

*Iodide of Potassium* is a remedy of deserved reputation in the treatment of some forms of syphilitic inflammation, especially in the later manifestations of the disease—in periostitis, ulceration of fauces and larynx, and in all the tertiary affections. It is also curative of periostitis and some cases of inflammation of bones when no syphilitic disease is present. In some inflammatory affections of the skin it is also manifestly useful.

*Digitalis, Veratrum viride* and all other arterial sedatives, have been employed in the treatment of inflammation; in pneumonia, acute rheumatism, typhoid fever, dysentery, puerperal peritonitis and all other diseases where the rapidity of the heart's action was greatly increased. That they act only as sedatives, reducing the frequency and force of the pulse seems probable; that this reduction in force and frequency is curative of inflammation is not certain; that it is productive of any beneficial influence may admit of doubt, though some of these medicines are prescribed with great confidence by the best informed practitioners. If one lung is rendered useless by inflammation, and the other is doing service for both, while the heart is aiding in the process by increased action, thus trying to compensate for the obstruction in the circulation and oxygenation of the blood, it is difficult to explain how any depressing effect upon it, can be productive of benefit. Clinical observation is referred to as sustaining the belief that it is useful. Facts are more stubborn than theory, and if such observations are *established*, satisfactory explanation will not long be wanting.

*Purgatives* are used in inflammation to free the bowels of morbid secretions and undue accumulations, and are of the first importance on account both of their direct and indirect influence. One of the most remarkable effects of purgation is often observed in elimination of urea or other constituents of the urine, in acute uræmia. The brain is relieved by purgation in some inflammatory affections. The liver is also thought to be and probably is, favorably effected by the indirect influence of purgatives. That they have been used more indiscriminately and more injuriously than most other remedies is probable; that they should not be wholly neglected is certain.

*Local remedies* are also employed in the treatment of inflammation. Local blood-letting naturally claims attention as the most powerful, direct and efficient remedy, and in some instances it appears reasonable to suppose that its effects are highly salutary. It is capable of emptying distended vessels and relieving the tension of tissues in external and local affections, but its controlling influence is not as apparent as the theory of its use is plausible; it is not as often necessary or as certainly useful as formerly supposed. Leeching and cupping are remedies having some objections which wholly or in part counteract their curative action, and it is well to inquire in the selection of these measures, if they will probably remove more inflammation than they are liable to cause.

*Cold* is a valuable remedy, supposed to act by constringing the uessels and interrupting the force of the blood current.

*Warmth* is often useful; it serves to allay pain, is supposed to soften the tissues and lessen engorgement by favoring exudation. Warmth is to be preferred in inflammations of the bowels, and within the chest; in rheumatism, erysipelas and in all cases likely to terminate in suppuration, since it is believed to quicken this process.

Among the local remedies for the treatment of inflammation we have blisters, sinapisms, turpentine, ammonia, croton oil, tartar emetic, issues, setons, moxa, all supposed to act upon the principle of revulsion, diverting the blood from diseased organs to those which were formerly healthy. In the early stages of inflammation these remedies are certainly quite inapplicable; they are supposed to be valuable in the later periods, serving to promote absorption of effusions; but that they are any time productive of more benefit than harm is not certain. The plan of counter-irritant treatment has been shamefully too common, and without denying its possible capacity for good, its manifest liability to do harm, is not to be forgotten.

Depressing treatment, in inflammation if unnecessary, is always injurious, and though a great majority of cases will recover if subjected to it; yet a still greater proportion might terminate thus favorably if left to themselves or treated by milder means, and earlier sustained and strengthened. Patients suffering from inflammation should certainly receive the support which exhausting disease will always cause the system to require. That much may be done to aid the vital power to overcome the most depressing influences cannot be doubted. Timely support, stimulation and relief from pain, will in all probability do much more than can be done by any of the depleting remedies. But it must not be carried over to the other extreme, and stimulation substituted as the prime object of cure. I have sometimes feared that physicians of the present day worked as much mischief by unnecessary, useless and injurious stimulation as did the physicians of former times by bleeding, blistering, purging, vomiting, mercury, antimony, low diet, and all the routine of unsanctified medication.

